# Microwave Dielectric Properties and Defect Behavior of xTiO_2_-(1-x)SiO_2_ Glass

**DOI:** 10.3390/ma18020320

**Published:** 2025-01-13

**Authors:** Chenyang Zhang, Sijian Gao, Mankang Zhu, Zhufeng Shao, Lanjian Nie, Hui Wang, Yanan Jia, Bo Fu

**Affiliations:** 1 China Building Materials Academy, Beijing 100024, China; 2 College of Materials Science and Engineering, Beijing University of Technology, Beijing 100124, China

**Keywords:** xTiO_2_-(1-x)SiO_2_ glass, dielectric constant, impedance analysis, oxide ion polarizability

## Abstract

xTiO_2_-(1-x)SiO_2_ (x = 2.9~8.2 mol%) glass specimens were synthesized using the flame hydrolysis technique. This study aimed to elucidate the influence of TiO_2_ incorporation on the optical characteristics, defect behavior, and microwave dielectric performance of these materials. UV–vis and near-infrared spectroscopic analyses were employed to investigate the hydroxyl and optical bandgap properties. Electron paramagnetic resonance (EPR) and AC impedance spectroscopy were utilized to examine oxygen vacancies, Ti^3+^ defects, and their respective behaviors. The findings revealed that, with increasing TiO_2_ content, the generation and migration of defects became more favorable, consequently leading to higher dielectric losses. The imaginary component of the electric modulus experimental data was fitted using the modified Kohlrausch–Williams–Watts (KWW) function, while the frequency-dependent AC conductivity was analyzed using the Jonscher power law. The calculated activation energy exhibited a decreasing trend with increasing TiO_2_ content, consistent with the characteristics of doubly ionized oxygen vacancies, suggesting the involvement of identical charge carriers in the relaxation and conduction mechanisms. Notably, the 8.2TiO_2_–91.8SiO_2_ glass specimen demonstrated exceptional microwave dielectric performance, exhibiting ε_r_ = 4.13, Q × f = 57,116 GHz, and τ_f_ = −4.32 ppm/°C, rendering it a promising candidate for microwave substrate applications.

## 1. Introduction

The dielectric properties of silicate glass materials have attracted considerable attention in the domains of communication and energy storage. Silicate glasses characterized by low dielectric constants, minimal dielectric loss, and low surface roughness are considered critical materials for next-generation 6G communication systems [1,2,3] and advanced packaging technologies [4,5]. Conversely, silicate glasses with high dielectric constants are highly promising for applications in high-energy-density capacitors and low-temperature co-fired ceramics (LTCC) [6]. Growing evidence suggests that the introduction of high field strength cations into silicate glasses can significantly modulate their dielectric properties [7], which has important implications for expanding material applications.

TiO_2_, a crucial intermediate oxide, is primarily employed to tailor properties such as thermal expansion, refractive index, and corrosion resistance in glass [8]. Recent studies have revealed that TiO_2_ incorporation can effectively increase the dielectric constant of silicate glass, but it also tends to enhance the crystallization propensity [9]. Additionally, current research on titanium silicate glasses typically requires the introduction of alkali and alkaline earth metals to modify their melting characteristics, which often results in higher dielectric losses in certain high-frequency applications. To simultaneously achieve the dual objectives of preventing TiO_2_ crystallization and reducing dielectric loss, we attempted to synthesize xTiO_2_-(1-x)SiO_2_ glass using the flame hydrolysis method. The glass was formed through the direct fusion of TiCl_4_ and SiCl_4_ gaseous precursors under high-temperature conditions using an oxyhydrogen flame. This approach effectively prevents TiO_2_ phase separation and crystallization while avoiding the introduction of network modifiers into the glass. Existing studies on xTiO_2_-(1-x)SiO_2_ glass prepared by this method have primarily focused on mechanical properties, thermal characteristics, and structural features [10] but lack systematic investigation into the relationship between microwave dielectric properties and material composition.

An often-overlooked factor influencing the dielectric performance of xTiO_2_-(1-x)SiO_2_ glass is oxygen volatilization. Extensive studies have demonstrated that oxygen vacancies can form during the high-temperature preparation process of SiO_2_ [11], acting as the primary intrinsic defects responsible for electrical conductivity in wide-bandgap materials. These oxygen vacancies play a pivotal role in controlling carrier concentration and interacting with dopants and impurities. Consequently, they are considered one of the principal factors contributing to high dielectric losses [12,13]. However, there is a significant knowledge gap regarding the defects and conduction mechanisms in xTiO_2_-(1-x)SiO_2_ glass, necessitating further in-depth investigations to elucidate these phenomena.

Through systematic modulation of xTiO_2_-(1-x)SiO_2_ glass composition, this study presents the first comprehensive investigation of how TiO_2_ content influences the microwave dielectric properties of xTiO_2_-(1-x)SiO_2_ glass. The primary research objectives are twofold: (1) to investigate the influence of varying TiO_2_ content on the microwave dielectric properties of the glass samples, and (2) to examine the glass defects as well as the relaxation and electrical conduction mechanisms based on comprehensive electron paramagnetic resonance (EPR) and alternating current (AC) impedance analyses. The systematic investigation aims to provide insights into the fundamental mechanisms governing the dielectric behavior of these glass systems, facilitating the development of advanced dielectric materials for various applications.

## 2. Experiment

In this study, following xTiO_2_-(1-x)SiO_2_ (in mol%) composition, seven sample groups were prepared using SiCl_4_ and TiCl_4_ as precursors via the flame hydrolysis process under identical melting temperature and oxidizing atmosphere conditions. The detailed material synthesis process can be found in the Supporting Information (as shown in Figure S1). The samples were labeled as S0 (x = 0), S1 (x = 2.9), S2 (x = 4.7), S3 (x = 5.7), S4 (x = 6.5), S5 (x = 7.3), and S6 (x = 8.2). The densities of the samples were determined using the Archimedes method. The optical absorption spectra of the samples were measured using an ultraviolet–visible near-infrared spectrophotometer (Lambda 950, PerkinElmer, Shelton, WA, USA). An electron paramagnetic resonance (A300-10/12, Bruker, Karlsruhe, Germany) spectrometer operating at a frequency of 9.45 GHz and a temperature of 77 K was employed to analyze the paramagnetic defects in the samples. The samples were processed and double-side polished into Φ20 × 0.7 mm pellets, with Pt electrodes deposited on both end faces. An impedance spectroscopy analyzer (Novocontrol Turnkey Concept 40 System, Novocontrol Technologies GmbH & Co. KG, Hundsangen, Germany) was utilized to determine the electrical properties of the samples, with a frequency range from 40 Hz to 1 MHz and a temperature range of 623–873 K.

The microwave dielectric properties were measured using the Hakki–Coleman method with a network analyzer (Agilent N5230A, Agilent Technologies, Santa Clara, CA, USA) and a temperature chamber. The temperature coefficient of the relative permittivity (*τ_f_*) in the temperature range of 293–353 K was calculated according to the following formula:

(1) $$\tau_{f}=\frac{f_{353}-f_{293}}{(353-293)\times f_{20}}\times 10^{6}$$

where $f_{353}$ and $f_{293}$ are the resonant frequencies at 353 K and 293 K, respectively.

## 3. Results and Discussion

### 3.1. Study of Spectral Properties

**Figure 1:**
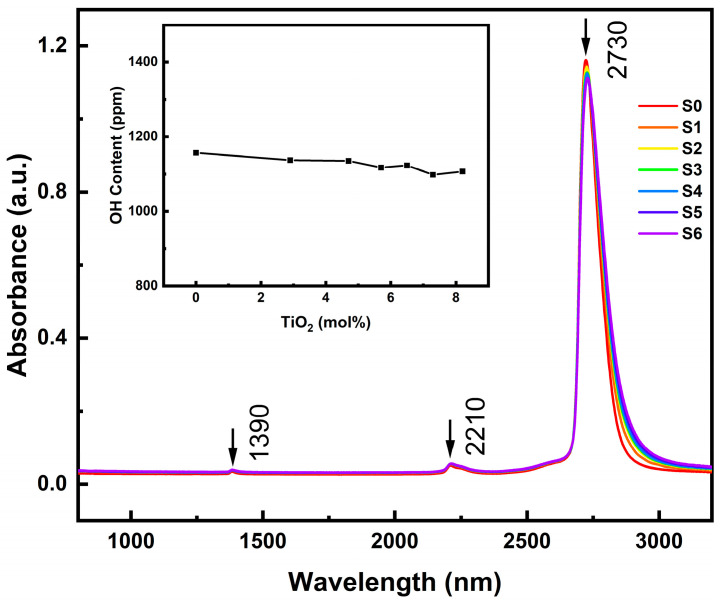
Infrared absorption spectra of samples S0–S6. The inset shows the magnified absorption peak at 2730 nm, and the table presents the calculated hydroxyl content values.

Figure 1 shows the infrared absorption spectra of the samples in the wavelength range of 800–3200 nm. Due to the preparation method, the xTiO_2_-(1-x)SiO_2_ glass samples exhibited a characteristic of high hydroxyl content, with distinct absorption peaks observed at 2730, 2210, and 1390 nm, which were consistent with the infrared absorption features of silica glass prepared using the flame hydrolysis method [14,15]. The results in Figure 1 reveal that as the TiO_2_ content increased, the characteristic absorption peak at 2730 nm exhibited a slight redshift, mainly due to the substitution of the larger Ti^4+^ ions for Si^4+^ ions, resulting in a decrease in the vibrational frequency of the hydroxyl groups. However, even with the introduction of up to 8 mol% TiO_2_, the overall structure of the silica network was not significantly affected. Based on the improved Beer–Lambert law [16,17], the hydroxyl content of the xTiO_2_-(1-x)SiO_2_ glass samples was calculated, and the results are shown in the inset of Figure 1. The results indicated that the hydroxyl content of all samples remained around 1100 ppm, suggesting that different TiO_2_ contents had almost no impact on the hydroxyl content in the glass due to the use of the same flame hydrolysis melting conditions.

**Figure 2:**
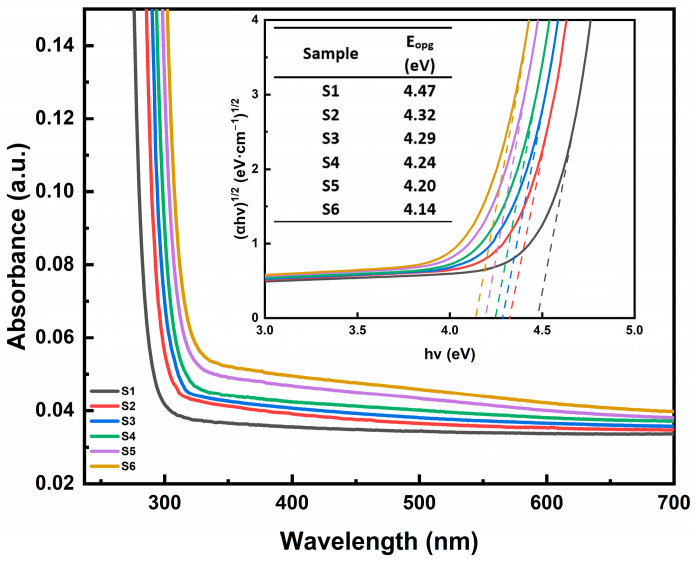
UV absorption spectra of samples S1–S6. The inset displays the Tauc plots, and the listed data are the indirect transition optical bandgap values obtained by extrapolation.

Figure 2 shows the UV–visible absorption spectra of different samples. TiO_2_ is a wide-bandgap semiconductor with a bandgap ranging from 3.0 to 3.2 eV, significantly influencing the UV absorption characteristics of SiO_2_ glass samples. An increase in TiO_2_ content will lead to a pronounced redshift of the UV absorption edge. Based on the Tauc plots (inset of Figure 2), the optical bandgap (*E*_opg_) values of the samples were calculated using the following formula:

(2) $$\alpha hv={C\left(hv-E_{opg}\right)}^{2}$$

Pronounced linear regions can be observed in each curve, indicating the validity of Equation (2) and showing that the optical bandgap originates from the amorphous absorption edge. By extrapolating the linear portions of the Tauc plots (the dashed line shown in the inset of Figure 2), the E_opt_ values are determined to decrease from 4.47 eV to 4.14 eV. Typically, the valence band of SiO_2_ is hybridized from O 2p and Si 3s orbitals, with an optical bandgap of around 8.5 eV [18]. The introduction of TiO_2_ significantly reduces the optical bandgap of the samples. The synergistic coupling effect between titanium ions and oxygen defect centers dominates the UV–visible absorption characteristics of xTiO_2_-(1-x)SiO_2_ glass: As Ti concentration increases, charge transfer transitions in the 155–248 nm range become more intense [19,20]. Simultaneously, oxygen vacancies and related defects form additional absorption centers at 200–300 nm [21], which synergistically amplify spectral changes with Ti valence state transitions. This mechanism results in a significant redshift of the UV absorption edge, leading to a reduced optical bandgap and broader absorption bands in the visible region (354–800 nm) [20].

### 3.2. Analysis of Defect Characteristics

#### 3.2.1. EPR

**Figure 3:**
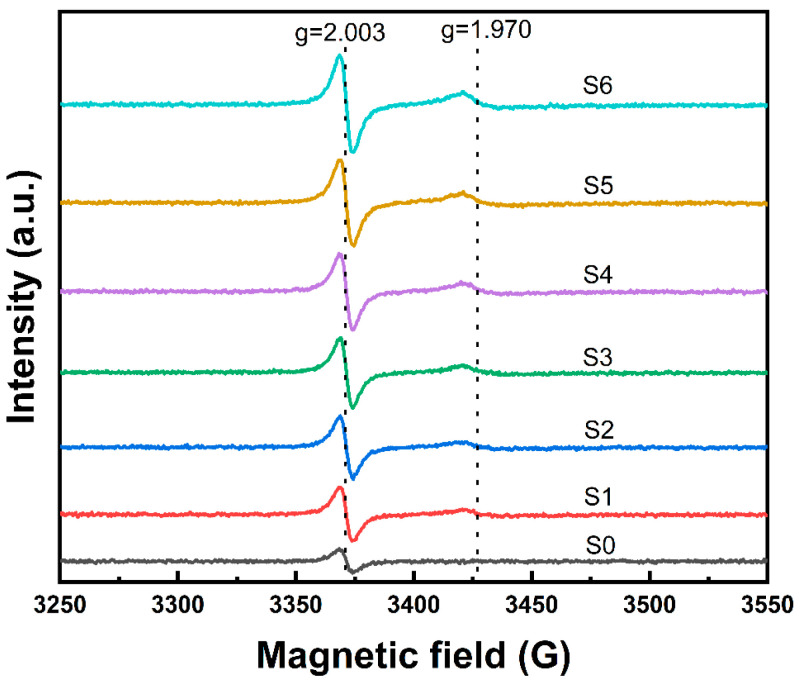
EPR spectrum of S0–S6 sample.

Electron paramagnetic resonance (EPR) is a powerful spectroscopic technique for studying materials with unpaired electrons, making it particularly effective for analyzing defects in glass. EPR’s high specificity and sensitivity make it an indispensable tool for investigating both intrinsic defects and transition metal ion behavior in glass, particularly in disordered systems where other techniques often fall short [22]. To investigate the influence of defects on the xTiO_2_-(1-x)SiO_2_ glass system, we studied the X-band EPR spectra characteristics of the samples at 77 K (as shown in Figure 3).

For the Ti-free reference sample S0, only one signal at g = 2.003 is observed, while the EPR spectra of samples S1–S6 exhibit two signals at g = 2.003 and g ≈ 1.970, with their intensities increasing with TiO_2_ content. The resonance peak at g = 2.003 corresponds to charge defects associated with oxygen atoms [23,24], whereas the peak at g ≈ 1.970 is related to the presence of paramagnetic Ti^3+^ centers [24]. The formation of these two defect structures is likely due to high-temperature dissociation during the flame hydrolysis synthesis of xTiO_2_-(1-x)SiO_2_ glass, leading to the generation of oxygen vacancies and the reduction of Ti^4+^ ions. The corresponding defect equations are:

(3) $$O_{O}\to V_{o}^{\cdot \cdot }+2e^{\prime }+\frac{1}{2}O_{2}$$

(4) $$Ti^{4+}+e^{\prime }\to Ti^{3+}$$

Furthermore, both EPR signals intensify with increasing TiO_2_ content, indicating that the introduction of TiO_2_ facilitates the formation of Ti^3+^ and oxygen vacancies in the glass network, which will lead to higher dielectric losses. To further investigate the defect behavior, we employed AC impedance spectroscopy to study the electrical properties and charge carrier transport characteristics of the xTiO_2_-(1-x)SiO_2_ system.

#### 3.2.2. Electric Modulus Analysis

The electric modulus represents the relaxation of the internal electric field within a material under conditions of constant electric displacement, and it is typically employed to characterize the intrinsic dielectric relaxation processes. Mathematically, the complex electric modulus is represented by the inverse of the complex dielectric permittivity [25,26]:

(5) $$M^{*}=M^{\prime }-jM^{\prime \prime }=\frac{1}{\varepsilon^{*}}=\frac{\varepsilon^{\prime }+j\varepsilon^{\prime \prime }}{{\left(\varepsilon^{\prime }\right)}^{2}+{\left(\varepsilon^{\prime \prime }\right)}^{2}}$$

The modulus formalism is applied to homogeneous systems, typically by modeling the modulus spectral response using a simplified *RC* element consisting of a resistor (*R*) and a capacitor (*C*) [27,28]:

(6) $$M^{\prime \prime }=\frac{\omega RC_{0}}{1+\omega^{2}R^{2}C^{2}}$$

**Figure 4:**
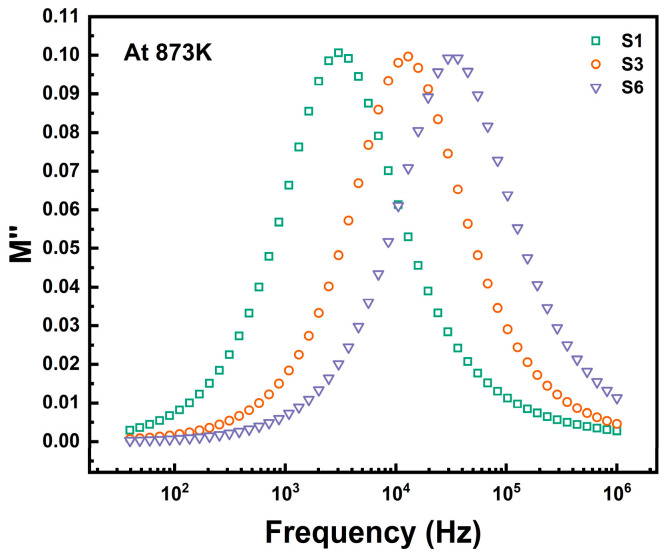
Frequency dependence of the imaginary part of the modulus of three samples, S1, S3, and S6 (873 K).

Figure 4 displays the frequency dependence of the imaginary part of the modulus ($M^{\prime \prime }$) for samples S1, S3, and S6 at 873 K. With increasing TiO_2_ content, the maximum value of $M^{\prime \prime }$ ($M_{max}^{\prime \prime }$) decreases slightly. At the peak frequency, $\omega_{p}=1/\tau =1/(RC)$, $M_{max}^{\prime \prime }=\frac{C_{0}}{2C}$ represents the minimum capacitance of the sample, where C_0_ is the vacuum capacitance. The center frequency $f_{max}$ corresponding to $M_{max}^{\prime \prime }$ shifts towards higher frequencies with increasing TiO_2_ content, indicating an enhancement of the thermally activated relaxation in the glass samples.

**Figure 5:**
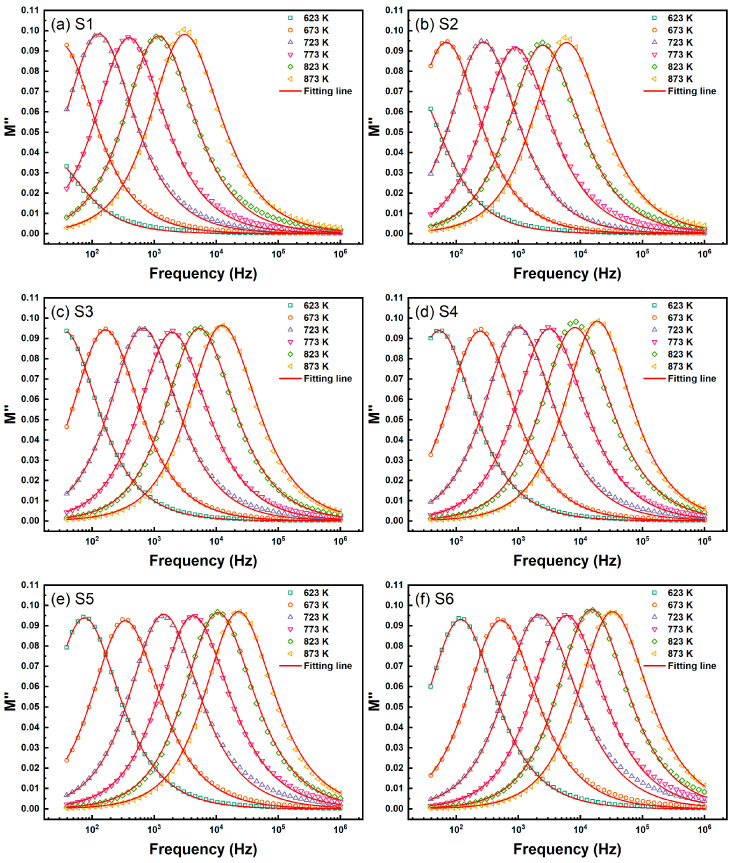
KWW fitting results for samples S1–S6.

The results in Figure 5 reveal that the $M_{max}^{\prime \prime }$ of samples S1–S6 remains essentially unchanged with increasing temperature. Consequently, the capacitance values of the samples are largely temperature independent. This implies that the dielectric constants of the samples do not vary significantly with temperature, indicating excellent temperature stability of the dielectric constant for all samples.

The $M^{\prime \prime }$ curves in Figure 5 exhibit an asymmetric lineshape, and this non-Debye behavior of the $M^{\prime \prime }$ spectra may be associated with the structural disorder characteristic of glass and the distribution of relaxation times arising from Coulombic interactions between differently charged species [29]. The degree of deviation from Debye-like relaxation within the material can be determined by the stretching exponent (*β*). By adopting the modified Kohlrausch–Williams–Watts (KWW) function proposed by Bergman to interpret the electric modulus behavior of glass systems, the imaginary part of the electric modulus is expressed as follows [30,31]:

(7) $$M^{\prime \prime }=\frac{M_{max}^{\prime \prime }}{(1-\beta )+\frac{\beta }{1+\beta }\left[\beta \left(\frac{f_{max}}{f}\right)+{\left(\frac{f}{f_{max}}\right)}^{\beta }\right]}$$

**Figure 6:**
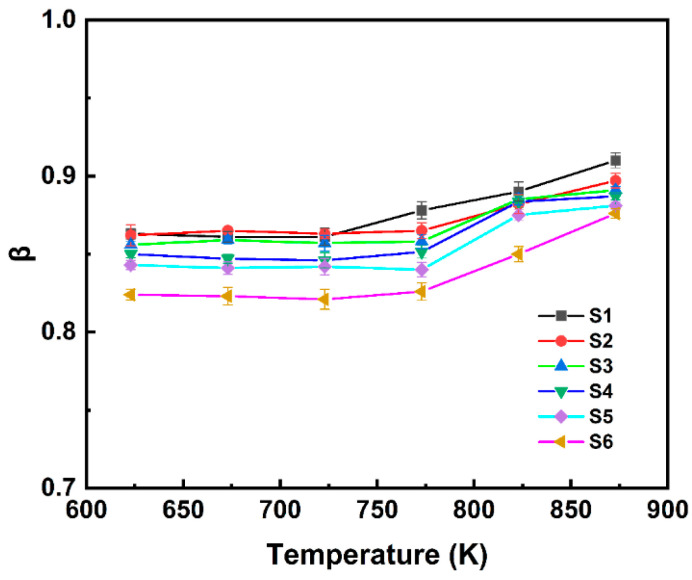
Temperature dependence of the exponent β for samples S1–S6.

The $M^{\prime \prime }$ data were fitted using Equation (7) (fitting results shown in Figure 5), and the obtained *β* values are summarized in Figure 6.

In the ideal Debye relaxation case, β approaches 1. When β < 1, this can be attributed to the existence of a distribution of relaxation times within the material. As shown in Figure 6, at the same temperature, the β value decreases with increasing TiO_2_ content (from S1 to S6). Because the β value correlates well with the average distance between charge carriers [32,33], the decreasing trend in β suggests that with increasing TiO_2_ content, the hopping distance between defect charge carriers decreases, leading to enhanced interactions among the charge carriers. Consequently, the relaxation process will exhibit more pronounced non-exponential behavior.

As shown in Figure 6, the variation in β with temperature exhibits a similar trend for each sample. In the temperature range of 623–723 K, the β values for different samples remained nearly constant. However, upon further increasing the temperature, the β values exhibited a certain degree of increase. This observation suggests that with rising temperature, the glass network becomes more relaxed, weakening the interactions between defect charge carriers and the surrounding matrix.

Based on the variation in the relaxation time $\tau_{M^{\prime \prime }}$ at different temperatures, shown in Figure 5, we calculated the relaxation activation energy W_d_ for different samples using Equation (8):

(8) $$\tau_{M^{\prime \prime }}=\tau_{0}exp\left(\frac{W_{d}}{kT}\right)$$

where $\tau_{M^{\prime \prime }}=\frac{1}{2\pi f_{max}}$, $\tau_{0}$ is the pre-exponential factor, *k* is the Boltzmann constant, *T* is the absolute temperature in Kelvin, and *W_d_* is the activation energy associated with the relaxation process.

**Figure 7:**
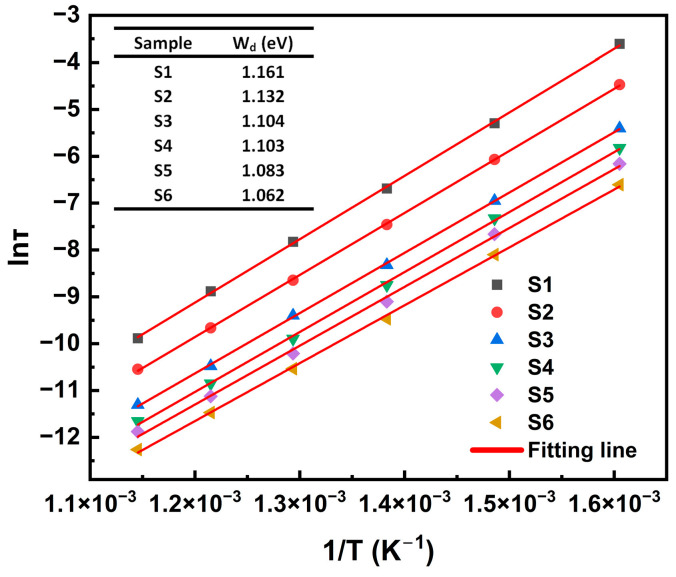
Arrhenius fitting of relaxation times for glass samples S1–S6.

Figure 7 displays the relationship between $ln(\tau_{M^{\prime \prime }})$ and the inverse temperature (1000/T), where the red solid lines represent the linear fitting results based on Equation (8). The results indicate that with increasing TiO_2_ content, the relaxation activation energy W_d_ decreases from 1.16 eV (S1) to 1.06 eV (S6), mainly due to the reduction in the binding ability of the glass matrix to defect charge carriers. It is generally accepted that oxygen vacancies are the main mechanism for charge-carrier conduction in silicate glass materials [34], with the activation energies for singly ionized or doubly ionized oxygen vacancies typically ranging from 0.3 to 0.4 eV and from 0.6 to 1.2 eV, respectively [35]. The relaxation activation energy results suggest that the relaxation mechanism in the presently studied glass system is consistent with the characteristics of doubly ionized oxygen vacancies.

#### 3.2.3. Analysis of AC Conductivity Characteristics

**Figure 8:**
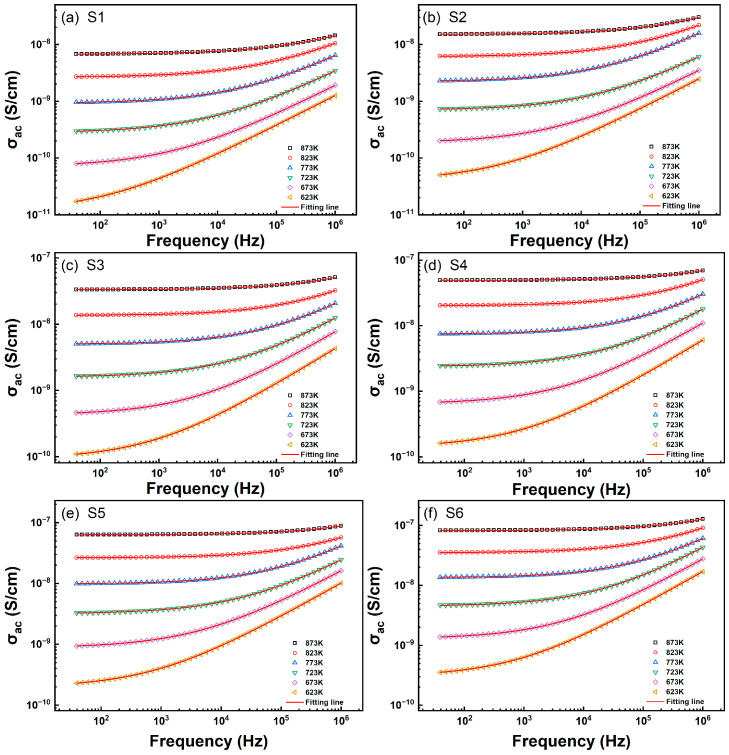
Here, (**a**–**f**) depict the conductivity curves for the S1–S6 glass samples, with the red curves representing the fitting results obtained using the Jonscher equation.

The transport behavior of charge carriers in glass can be determined through AC conductivity. Figure 8a–f illustrate the frequency-dependent conductivity behavior of glass systems S1–S6 at different temperatures.

**Figure 9:**
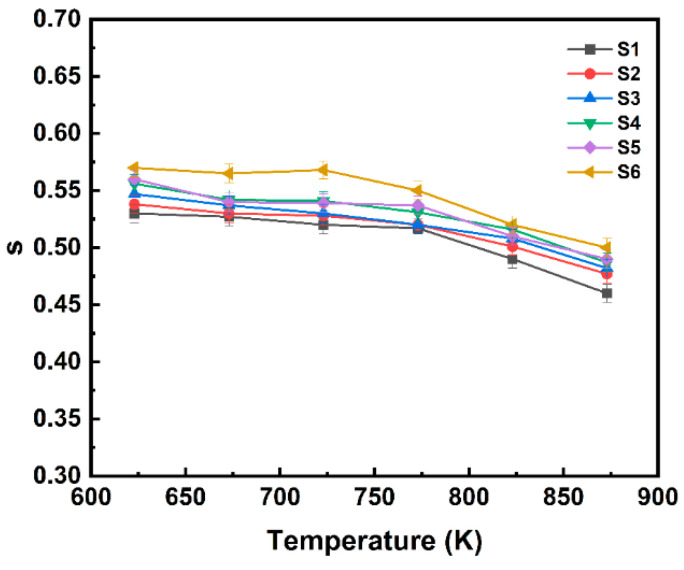
The temperature dependence of the Jonscher power law exponent (s) for the S1–S6 glass samples.

We performed a non-linear curve fitting for the conductivity data using the Jonscher power law, which can be expressed as $\sigma =\sigma_{dc}+A\omega^{s}$. Here, $\sigma_{dc}$ represents the DC conductivity, while the coefficient A and exponent s are related to the temperature and material properties. Generally, s serves as a measure of the degree of interaction between the charge carriers and their environment, influenced by the distribution of cluster sizes and their relaxation rate distributions. Sidebottom’s studies [36,37] indicate that the exponent s depends on the dimensionality of the local conduction space and increases with increasing dimensionality. The results depicted in Figure 9 show that, under the same temperature conditions, the exponent s increases with increasing TiO_2_ concentration, which may be a consequence of an increase in the dimensionality of the conductive space. Moreover, the values of s for all samples decrease with increasing temperature (as shown in Figure 9), indicating that the correlated barrier hopping (CBH) model is suitable for explaining the charge transport mechanism in these samples. According to this model, the AC conductivity in all samples arises from thermally activated charge carriers hopping between two sites by overcoming the potential barriers separating them [35].

**Figure 10:**
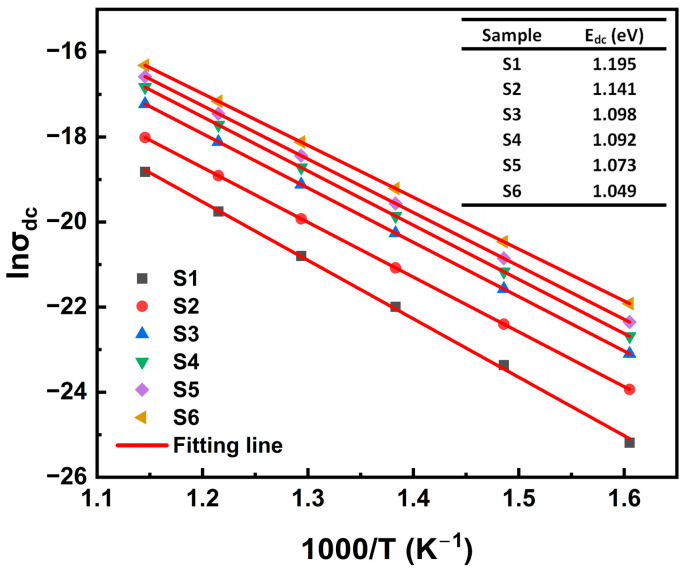
The calculation of the activation energies for DC conductivity of the S1–S6 glass samples. The solid lines in the figure represent the Arrhenius fitting results.

Figure 10 shows the variation in $ln\left(\sigma_{DC}\right)$ as a function of 1000/T. Based on these results, we calculated the activation energies for the DC conductivity of the samples using the Arrhenius law, and they lay in the range of 1.049–1.195 eV. It is noteworthy that the values of the activation energies for relaxation and conductivity are similar, indicating that the relaxation and conduction processes correspond to the same mechanism, involving the same charge carriers (doubly ionized oxygen vacancies). The increase in TiO_2_ content enhances the probability of defect formation and migration in the xTiO_2_-(1-x)SiO_2_ glass system, leading to higher conductivity and a reduction in the conductivity activation energy, which, in turn, increases the conductivity loss which is a component of the dielectric loss. Therefore, we speculate that this may reduce the Q × f value of the material.

### 3.3. Microwave Dielectric Properties

**Figure 11:**
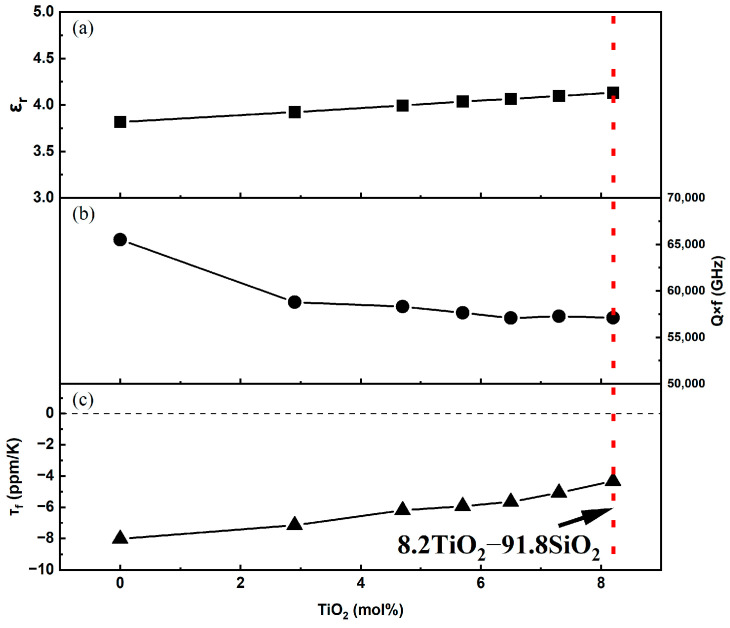
Microwave dielectric properties of the xTiO_2_-(1-x)SiO_2_ glass system. (**a**) Dielectric constant ($\varepsilon_{r}$), (**b**) quality factor (Q × f), (**c**) temperature coefficient of resonant frequency (*τ_f_*).

Figure 11 presents the microwave dielectric performance of the xTiO_2_-(1-x)SiO_2_ glass samples. As shown in Figure 11a, the permittivity (ε_r_) of the samples exhibits an approximately linear increase with increasing TiO_2_ content. Furthermore, the dielectric constants of the samples are lower than those reported for xTiO_2_-(1-x)SiO_2_ ceramic materials by Li and Hu [38,39] (as shown in Table 1), which is consistent with the general understanding that the formation of the glassy phase reduces the dielectric constant of the material [40]. The smaller radius, higher charge state, and larger polarizability of Ti^4+^ ions facilitate ionic polarization. The increasing amount of introduced TiO_2_ increases the number of polarizable units, consequently leading to a moderate increase in the dielectric constant.

Based on the Clausius–Mosotti (CM) equation, the polarizability ($\alpha_{D}$) of different samples can be calculated from their dielectric constant ($\varepsilon_{r}$) and molar volume ($V_{m}$):

(9) $$\alpha_{D}=\frac{1}{b}V_{m}\frac{\varepsilon_{r}-1}{\varepsilon_{r}+2}$$

where *b* = 4π/3, and the molar volume ($V_{m}$) is determined by the molecular weight (MW) of the glass, its density (*ρ*), and the Avogadro constant ($N_{A}$):

(10) $$V_{m}=\frac{MW}{\rho N_{A}}$$

It is noted that the density of the xTiO_2_-(1-x)SiO_2_ glass material is nearly independent of the titanium content, which is consistent with Shelby’s earlier results [16]. The calculated values of *ρ* and $V_{m}$ are summarized in Table 2. The calculation results indicate that the increase in the content of Ti ions with high polarizability leads to a continuous increase in the polarizability $\alpha_{D}$ value (as shown in Table 2). Furthermore, based on the following equation, we calculated the polarizability of oxygen ions ($\alpha_{O^{2-}}$) for samples with different titanium contents:

(11) $$\alpha_{O^{2-}}=\frac{\alpha_{D}-\alpha_{c}}{X}$$

(12) $$X=\sum_{i}x_{i}y_{i}$$

(13) $$\alpha_{c}=\sum_{i}x_{i}\alpha_{i}^{c}$$

where $x_{i}$ is the mole fraction of each component, $y_{i}$ is the number of oxygen ions in component *i*, and X is the total mole number of oxygen ions in the entire glass. $\alpha_{i}^{c}$ is the cation polarizability of component *i*, and $\alpha_{c}$ is the sum of all cation polarizabilities.

**Figure 12:**
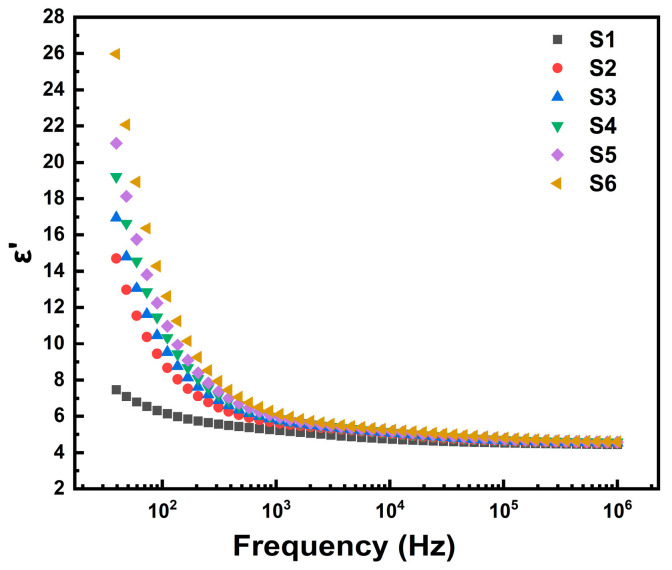
Frequency spectrum curves of the real part of the dielectric constant for samples S1–S6 at 873 K.

The α(Si^4+^) and α(Ti^4+^) values are cited from Shannon’s ionic polarizability theory [41]. The calculated $\alpha_{O^{2-}}$ values for samples with different titanium contents are shown in Table 2. It is observed that the $\alpha_{O^{2-}}$ polarizability increases with increasing titanium content, and the values are significantly higher than the theoretical polarizability value of $\alpha_{O^{2-}}$ (2.01 Å^3^). Combining the aforementioned trends in relaxation and electrical conductivity activation energy, the constraining ability of the glass matrix on defects gradually decreases, ultimately leading to an increase in the polarizability of $\alpha_{O^{2-}}$. When variations in TiO_2_ content lead to changes in oxygen ion polarizability, the frequency response characteristics of oxygen ions change accordingly, resulting in alterations in the spectral behavior of the sample’s dielectric constant. Generally, ions with higher polarizability exhibit more pronounced relaxation behavior in response to frequency variations, manifesting as more significant dielectric dispersion behavior. Our experimental results demonstrate that with increasing TiO_2_ content, the overall polarizability of the samples increases, particularly the oxygen ion polarizability, leading to more pronounced dielectric dispersion behavior, as shown in Figure 12. This means the relaxation behavior with frequency variation becomes more evident. These findings validate the rationality of our calculations.

The relationship between the quality factor (Q × f) of the samples and the TiO_2_ content is shown in Figure 11b. As the value of x increases from 0 to 8.2 mol%, the Q × f value of the samples decreases from 65,517 GHz to 57,116 GHz (a decrease of 12.8%). This is mainly due to the formation of oxygen vacancies and related defects under high-temperature conditions, which has been verified by the aforementioned EPR and AC impedance analysis results. The presence of oxygen vacancies will enhance the anharmonic vibrations between the phonon system and the alternating electric field, thereby increasing the extrinsic dielectric loss [42]. With increasing TiO_2_ concentration, the formation of more oxygen vacancies leads to higher polarization loss and conductivity losses, which is the primary cause of the decreased Q × f value of the glass.

The results in Figure 11c show that the samples exhibit relatively low negative values for the resonant frequency temperature coefficient, and as the TiO_2_ content increases, the τ_f_ value increases from −7.15 ppm/K to −4.32 ppm/K (S1 to S6), which is closer to 0 ppm/K compared to the τ_f_ value of SiO_2_ (approximately −8.0 ppm/K). These results also validate the good dielectric constant temperature stability exhibited by the samples in the AC impedance spectroscopy results.

## 4. Conclusions

In this study, the optical properties, microwave dielectric performance, and defect behavior of the xTiO_2_-(1-x)SiO_2_ glass series synthesized via the flame hydrolysis method were investigated. The optical bandgap of the samples was found to decrease significantly with increasing TiO_2_ content, as detected by UV–visible spectroscopy. The defect behavior of the materials was studied through EPR and AC impedance analysis, where the activation energies obtained from the relaxation and conduction processes of all samples were highly consistent and corresponded to the characteristics of doubly ionized oxygen vacancies. With increasing TiO_2_ content, the formation and migration of defects became more favorable, leading to higher dielectric losses. Furthermore, AC impedance spectroscopy revealed the temperature stability of the dielectric constant. The calculation of the microwave dielectric constant based on the Clausius–Mossotti equation indicated that, as the titanium content increased, the degree of glass network polymerization decreased and the $\alpha_{O^{2-}}$ polarizability increased. The 8.2TiO_2_-91.8SiO_2_ glass sample exhibited excellent microwave dielectric performance (ε_r_ = 4.13, Q × f = 57,116 GHz, τ_f_ = −4.32 ppm/°C), demonstrating its promising potential in applications as a microwave substrate material.

## Figures and Tables

**Table 1:** Comparative analysis of properties between the present study and data in the literature.

Sample	$\varepsilon_{r}$	Q × f (GHz)	*τ_f_* (ppm/K)	Reference
S6	4.13	57,116	−4.32	This work
Silica glass	3.82	75,000	−8.0	
0.84SiO_2_-0.16TiO_2_	5.91	39,680	−4.53	[38]
0.85SiO_2_-0.15TiO_2_	5.4	40,500	2.5	[39]

**Table 2:** Summary of dielectric constants $\varepsilon_{r}$, molar volumes $V_{m}$, and oxygen ion polarizability $\alpha_{O^{2-}}$ parameters for the xTiO_2_-(1-x)SiO_2_ glass system.

Sample	Density (g/cm^3^)	Molar Volume (Å^3^)	$\varepsilon_{r}$	$\alpha_{D}$	*α* (*O*^2−^)
S0	2.200	45.35	3.83	5.26	2.63
S1	2.202	45.74	3.94	5.41	2.67
S2	2.203	45.99	4.00	5.50	2.70
S3	2.201	46.18	4.04	5.55	2.71
S4	2.203	46.25	4.07	5.59	2.72
S5	2.208	46.27	4.10	5.63	2.74
S6	2.207	46.43	4.13	5.68	2.75

## Data Availability

The original contributions presented in this study are included in the article. Further inquiries can be directed to the corresponding author(s).

## Supplementary Materials

The following supporting information can be downloaded at: https://www.mdpi.com/article/10.3390/ma18020320/s1, Figure S1: Schematic diagram of the flame hydrolysis process process.
