# Supplementary material for: Microwave Dielectric Properties and Defect Behavior of xTiO2-(1-x)SiO2 Glass
Source: Materials (Basel). 2025 Jan 13;18(2):320. doi: 10.3390/ma18020320 (PMC11766635; doi:10.3390/ma18020320)
Supplement: Supplementary file 1 [file materials-18-00320-s001.zip › materials-3362646-supplementary.pdf]

Supplementary materials

Microwave Dielectric Properties and Defect Behavior of $xTiO_2$-(1-x)SiO_2 Glass

Chenyang Zhang [1,*], Sijian Gao [1], Mankang Zhu [2], Zhufeng Shao [1], Lanjian Nie [1], Hui Wang [1], Yanan Jia [1] and Bo Fu [1]

1 China Building Materials Academy, Beijing 100024, China
2 College of Materials Science and Engineering, Beijing University of Technology, Beijing 100124, China
* Correspondence: zcycbma@163.com

The detailed synthesis procedure

The following details the synthesis procedure, which has been included as supplementary material to the manuscript.

During the glass deposition process, gaseous precursors $SiCl_4$ and $TiCl_4$ were mixed and transported to the furnace chamber using O_2 as the carrier gas, where they underwent oxidation and hydrolysis reactions with the oxyhydrogen flame. The reactions occurring throughout this process are as follows:

$$2H_2(g)+O_2(g) \rightarrow 2H_2O(g) \quad \text{(S1)}$$

$$SiCl_4(g)+(n+2)H_2O(g)\rightarrow SiO_2{\cdot}nH_2O(s)+4HCl(g) \quad \text{(S2)}$$

$$SiCl_4(g)+O_2(g)\rightarrow SiO_2(s)+Cl_2(g) \quad \text{(S3)}$$

$$TiCl_4(g)+(n+2)\ H_2O(g)\rightarrow TiO_2{\cdot}nH_2O(s)+4HCl(g) \quad \text{(S4)}$$

$$TiCl_4(g)+O_2(g)\rightarrow TiO_2(s)+2Cl_2(g) \quad \text{(S5)}$$

$$H_2(g)+Cl_2(g) \rightarrow 2HCl(g) \quad \text{(S6)}$$

To ensure complete oxidation of the samples, O_2 was utilized to transport the vaporized $SiCl_4$ and $TiCl_4$ precursors. The primary chemical reactions of the gaseous $SiCl_4$ and $TiCl_4$ precursors in the high-temperature furnace environment were S3 and S5. Concurrent with these reactions, the formed SiO_2 and TiO_2 molecular clusters underwent collision and aggregation to form nanoparticles, which subsequently vitrified under high-temperature conditions, ultimately forming $xTiO_2$-(1-x)SiO_2 glass.

Figure S1 illustrates the schematic diagram of the current deposition system. The deposition equipment consists of four main components: 1) furnace chamber with associated rotational lifting mechanism, 2) burner, 3) hydrogen and oxygen flow control system, and 4) $SiCl_4$ and $TiCl_4$ vaporization and flow control system. During the deposition process, parameters of these four components were regulated to maintain process stability.

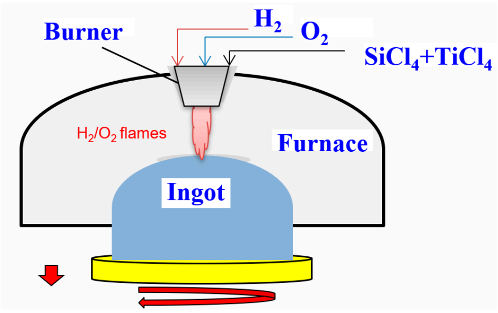

Figure S1. Schematic diagram of the flame hydrolysis process process.
